# Low Frequency of Infection with Avian Influenza Virus (H5N1) among Poultry Farmers, Thailand, 2004

**DOI:** 10.3201/eid1403.070662

**Published:** 2008-03

**Authors:** Soawapak Hinjoy, Pilaipan Puthavathana, Yongjua Laosiritaworn, Khanchit Limpakarnjanarat, Phisanu Pooruk, Teerasak Chuxnum, James M. Simmerman, Kumnuan Ungchusak

**Affiliations:** * Ministry of Public Health, Nonthaburi, Thailand; † Mahidol University, Bangkok, Thailand; ‡ Global Disease Detection/International Emerging Infections Program, Nonthaburi, Thailand

**Keywords:** Avian influenza, poultry exposure, microneutralization, dispatch

## Abstract

In Thai provinces where avian influenza outbreaks in poultry had been confirmed in the preceding 6 months, serum from 322 poultry farmers was tested for antibodies to avian influenza virus subtype H5N1 by microneutralization assay. No study participant met the World Health Organization serologic criteria for confirmed infection.

During late 2003 and 2004, highly pathogenic avian influenza virus (H5N1) caused extensive outbreaks and die-offs in poultry flocks in Thailand and several other countries in Southeast Asia (*1*). From January through March 2004, 12 cases, 8 fatal, in humans resulted from infection with influenza virus (H5N1) in Thailand (*2*). In response, the Thailand Department of Livestock Development enlisted government employees to conduct a large-scale cull of poultry in the affected provinces (www.dld.go.th/home/bird_flu/emergency.html). This effort began on January 23, 2004, and resulted in the slaughter of >21 million birds (www.fao.org/ag/againfo/subjects/en/health/diseases-cards/avian_bg.html). Poultry farmers and persons involved in culling are at increased risk for infection (*3*). In May 2004, we conducted a seroepidemiologic investigation of Thai poultry farmers to determine the frequency of avian influenza (H5N1) transmission to humans.

## The Study

We conducted a cross-sectional study among poultry farmers and cullers from 1 district in each of the 5 provinces (Chachoengsao, Kanchanaburi, Khon Kaen, Sukhothai, and Suphanburi) where outbreaks of avian influenza (H5N1) among poultry and human infections had been confirmed since January 2004 (Figure). With the assistance of provincial human and animal health authorities, we contacted farmers living in these districts. Informed consent was obtained, and a brief interview was conducted. Because the precise timing of potential exposures could not be determined, a single serum sample was collected from each patient and stored at –20°C until tested under Biosafety Level 3 (BSL-3) conditions. Specimens were tested, according to adapted methods described by Katz et al. (*4*), at the Department of Microbiology, Faculty of Medicine, Siriraj Hospital, Mahidol University by Microneutralization assay (micro-Nt) for antibody to H5N1 viruses. Before this study, senior laboratory staff from Siriraj Hospital received 2 weeks of on-site training by a visiting scientist from the US Centers for Disease Control and Prevention who had expertise with this assay. The World Health Organization (WHO) defines a positive test result as a microneutralization antibody titer for influenza virus (H5N1) of >80 with a confirmatory ELISA or Western blot assay (3,4) (www.who.int/csr/disease/avian_influenza/guidelines/case_definition2006_08_29/en/index.html). Serum samples from persons >50 years of age were excluded from laboratory analysis because the microneutralization assay for antibodies against subtype H5N1 has been reported to be less specific for older persons (*5*).

**Figure:**
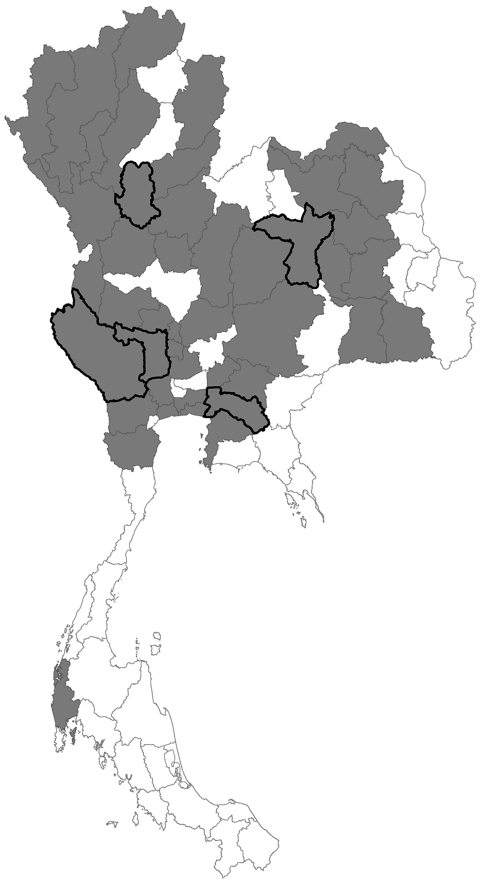
Map of Thailand. Gray shading indicates provinces with confirmed avian influenza outbreaks; black outlines indicate provinces included in this study.

Of 350 farmers asked to participate, 322 (92%) enrolled in the study, of which 167 (52%) were women, and 28 (8%) persons declined to participate. The mean age of participants was 34 years (range 5–50 years) (Table). Among participants, 188 (58%) reported handling sick or dying poultry, 107 (33%) were involved in culling operations of apparently well poultry in outbreak areas, and 27 (9%) reported only contact with well poultry in the context of routine farming practices. Although no study participant had an anti-H5N1 antibody titer of >80, 7 (2.2%) farmers had lower reactive antibody titers. Of these, 4 had titers of 10, 2 had titers of 20, and 1 had a titer of 40. The small number of study participants with anti-H5 antibody titers precluded statistical comparisons to those without reactive antibodies.

**Table:** Characteristics of 322 persons living on poultry farm in areas where  avian influenza (H5N1) infections among poultry and humans had been confirmed  since January 2004, Thailand

Variable	No. persons (%)
Province	
Chachoengsao	61 (18.9)
Kanchanaburi	32 (9.9)
Khon Kaen	65 (20.2)
Sukhothai	84 (26.1)
Suphanburi	80 (24.8)
Sex	
M	155 (48.1)
F	167 (51.9)
Age, y*	
<10	15 (4.7)
11–20	32 (9.9)
21–30	49 (15.2)
31–40	121 (37.6)
41–50	105 (32.6)
Current smokers	67 (20.8)
Chronic illness	74 (23.0)
Type of poultry maintained†	
Layer hen	111 (34.5)
Broiler	42 (13.0)
Fighting cock	88 (27.3)
Backyard chicken	89 (27.6)
Egg-laying duck	7 (2.2)
Meat duck	8 (2.5)
Ornamental birds	3 (0.9)
Type of poultry farm	
Company farm	125 (38.8)
Individual farm (backyard)	197 (61.2)
Observed increased deaths of poultry	231 (71.7)
Living on a mixed swine/poultry farm	24 (7.5)

*Age range 5–50 y; median 35 y. †Not mutually exclusive.

## Conclusions

Poultry farmers and cullers are at increased occupational risk for exposure to avian influenza viruses. However, since 2004, infections have been less commonly reported in cullers, while poultry farmers have made up a large proportion of cases worldwide. A study in Hong Kong Special Administrative Region, People’s Republic of China, examined influenza virus (H5N1) transmission and risk factors among poultry workers and government workers involved in culling during the 1997–98 outbreak (*3*). The study concluded that although no hospitalized poultry workers were identified among the 18 patients in that outbreak, 3% of 293 cullers and 10% of 1,525 poultry workers had antibody titers against influenza (H5N1) of >80, which suggested that a substantial number of mild or asymptomatic infections had occurred in this occupationally exposed population. In contrast, we found that no poultry workers had microneutralization titers >80, whereas 7 (2%) had lower titers that did not meet the WHO definition for seropositivity.

These findings could have several possible explanations. The lower titers may have resulted from cross-reactivity with circulating antibodies after previous human influenza virus infections (*5**,**6*). These low titers could be the result of mild or asymptomatic influenza (H5N1) infections because not all influenza virus infections invariably result in marked antibody responses (*7*). Likewise, these results could reflect the decay of antibody titers over time (*8*). Finally, the Micro-NT assay is a highly specific and strain-sensitive test. Although we used the same virus that was circulating in Thailand at that time, these lower titers could be attributable to infections with another virus variant.

Most human influenza (H5N1) infections have occurred in persons who had had direct contact with sick or dying poultry (*9*–*11*). While human infections with avian influenza (H5N1) continue to be reported, growing evidence indicates that this virus is not easily transmitted from poultry to humans and that mild or asymptomatic infections in humans are not common. A seroepidemiologic investigation in rural Cambodia surveyed 351 participants from 93 households in an area where influenza (H5N1) infections in poultry and a single fatal human case had been documented (*10*). Despite frequent, direct contact with poultry suspected of having influenza (H5N1) infection, none of the Cambodian study participants had antibodies reactive to this subtype. A similar study in Nigeria found that all of 295 poultry workers had negative test results for influenza (H5N1) neutralizing antibodies (*12*). Studies of healthcare workers suggest that transmission of influenza virus (H5N1) to hospital staff who cared for infected patients also appears to be uncommon (*13**–**15*).

Our study provides additional evidence to suggest that influenza virus (H5N1) is not easily transmitted to humans. However, the wide geographic distribution of this subtype, ubiquitous exposures, and the high case-fatality ratio from the infection underscore the importance of adherence to poultry-handling practices recommended by the Food and Agriculture Organization and WHO (www.wpro.who.int/NR/rdonlyres/7693BAF7-13E7-42DB-B92B-004CF5D517E7/0/WHOinterimrecommendation26012004.pdf, www.fao.org/ag/againfo/subjects/en/health/diseases-cards/avian_qa.html#8). Molecular surveillance indicates that the avian influenza virus (H5N1) continues to evolve rapidly (www.who.int/csr/disease/avian_influenza/guidelines/recommendationvaccine.pdf). Additional seroepidemiologic studies are warranted to monitor for changes in transmissibility and the spectrum of clinical illness.
